# Mechanistic Multilayer Quantitative Model for Nonlinear Pharmacokinetics, Target Occupancy and Pharmacodynamics (PK/TO/PD) Relationship of D-Amino Acid Oxidase Inhibitor, TAK-831 in Mice

**DOI:** 10.1007/s11095-020-02893-x

**Published:** 2020-08-05

**Authors:** Tomoki Yoneyama, Sho Sato, Andy Sykes, Rosa Fradley, Stuart Stafford, Shyam Bechar, Eimear Howley, Toshal Patel, Yoshihiko Tagawa, Toshiya Moriwaki, Satoru Asahi

**Affiliations:** 1 Present Address: Drug Metabolism and Pharmacokinetics Research Laboratories, Takeda Pharmaceutical Company Limited, Fujisawa, Kanagawa Japan; 2 Drug Metabolism and Pharmacokinetics Research Laboratories, Takeda Cambridge Ltd, Cambridge, UK; 3 Pharmacology, Takeda Cambridge Ltd, Cambridge, UK

**Keywords:** D-amino acid oxidase inhibitor, PK/PD, multilayer quantitative model, TAK-831, target occupancy

## Abstract

**Purpose:**

TAK-831 is a highly selective and potent inhibitor of D-amino acid oxidase (DAAO) currently under clinical development for schizophrenia. In this study, a mechanistic multilayer quantitative model that parsimoniously connects pharmacokinetics (PK), target occupancy (TO) and D-serine concentrations as a pharmacodynamic (PD) readout was established in mice.

**Methods:**

PK, TO and PD time-profiles were obtained in mice and analyzed by mechanistic binding kinetics model connected with an indirect response model in a step wise fashion. Brain distribution was investigated to elucidate a possible mechanism driving the hysteresis between PK and TO.

**Results:**

The observed nonlinear PK/TO/PD relationship was well captured by mechanistic modeling framework within a wide dose range of TAK-831 in mice. Remarkably different brain distribution was observed between target and reference regions, suggesting that the target-mediated slow binding kinetics rather than slow penetration through the blood brain barrier caused the observed distinct kinetics between PK and TO.

**Conclusion:**

A quantitative mechanistic model for concentration- and time-dependent nonlinear PK/TO/PD relationship was established for TAK-831 in mice with accounting for possible rate-determining process. The established mechanistic modeling framework will provide a quantitative means for multilayer biomarker-assisted clinical development in multiple central nervous system indications.

**Electronic supplementary material:**

The online version of this article (10.1007/s11095-020-02893-x) contains supplementary material, which is available to authorized users.

## Introduction

TAK-831 (4-hydroxy-6-{2-[4-(trifluoromethyl)phenyl]ethyl}pyridazin-3(2*H*)-one) is a highly selective and potent inhibitor of D-amino acid oxidase (DAAO). DAAO is a peroxisomal enzyme active towards neutral D-amino acids, and has been linked to the metabolism of D-serine. DAAO is mainly expressed in liver and kidney but is atowards neutral D-aminolso enriched in the central nervous system (CNS) (1,2). Within the mammalian brain, DAAO activity is absent or scarce in the forebrain and is confined to the brain stem and cerebellum (3,4). DAAO expression in whole blood is low. DAAO inhibition elevates D-serine in the cerebellum (5,6). D-serine has been demonstrated to be a co-agonist of *N*-methyl-D-aspartate (NMDA) glutamate receptors that, along with glutamate, mediates NMDA receptor transmission, synaptic plasticity and other physiological functions. D-serine is also a known endogenous ligand for the delta (δ)2 glutamate receptor (GluRδ2), which has been implicated in synaptic plasticity and long-term depression (5). TAK-831 has shown to increase D-serine levels in the cerebellum of normal mice and demonstrated a positive effect in mouse models associated with negative symptoms and cognitive impairment in schizophrenia (7). In the novel object recognition test in mice, animals were able to identify the novel object when treated with a range of acute and chronic doses of TAK-831. In the delayed paradigm of conditioned eyeblink behavior in mice, a model of cerebellar-based associative learning, TAK-831 showed improvement in the acquisition of the conditioned response and in reversing the scopolamine induced deficit. TAK-831 was also efficacious in the Balb/c social interaction model, a model of negative symptoms. Based on the evidence above, TAK-831 is currently under development for the treatment of cognitive impairment associated with schizophrenia (CIAS), and negative symptoms of schizophrenia (8).

Through years of implementation in drug discovery and development, pharmacokinetics (PK)/pharmacodynamics (PD) modeling has demonstrated tremendous values in elucidating the quantitative relationship between the PK of a therapeutic intervention and the resulting PD effects (9). However, the application of PK/PD modeling to CNS indications has been very modest compared with other therapeutic areas such as oncology, inflammation and metabolic disorders (10–12). Possible explanations include the lack of translationally suitable preclinical animal models, the subjective nature of many clinical endpoints and the pharmacological and pathophysiological complexity of CNS diseases. Therefore, mechanistic PK/PD modeling incorporating translationally applicable multilayer biomarkers is of crucial importance to better understand the quantitative relationship between PK, target engagement and downstream biological pathway modulation especially in CNS drug discovery and development.

The PK/ PD model analysis of DAAO inhibitors has been performed by Strick et.al. (4). In their study, while the relationship between free brain concentration of DAAO inhibitors and D-serine concentration in cerebellum was analyzed in mice, the relevance of target occupancy (TO) in plasma PK and cerebellum PD was not investigated. Since TO is a measurable biomarker in humans by positron emission tomography (PET) technology (13,14), the evaluation of TO including time-course profiling in pharmacological model animals is deemed meaningful.

The purpose of the present study was to establish a quantitative mechanistic model framework that could describe the relationship between PK, TO and PD after an oral administration of TAK-831 in mice. Since a time-lag was observed between plasma PK and cerebellum TO in mice, the concentration of TAK-831 in cerebellum (target region) and front part of cerebrum (reference region) was investigated. The established quantitative model framework was found useful to quantitatively understand the nonlinear PK/TO/PD relationship and their dose- and time-dependency around pharmacologically efficacious doses in mice. The model translation to humans and subsequent application to multilayer biomarker-assisted clinical development of TAK-831 is also discussed.

## Materials and Methods

### Chemicals and Reagents

**Fig. 1:**
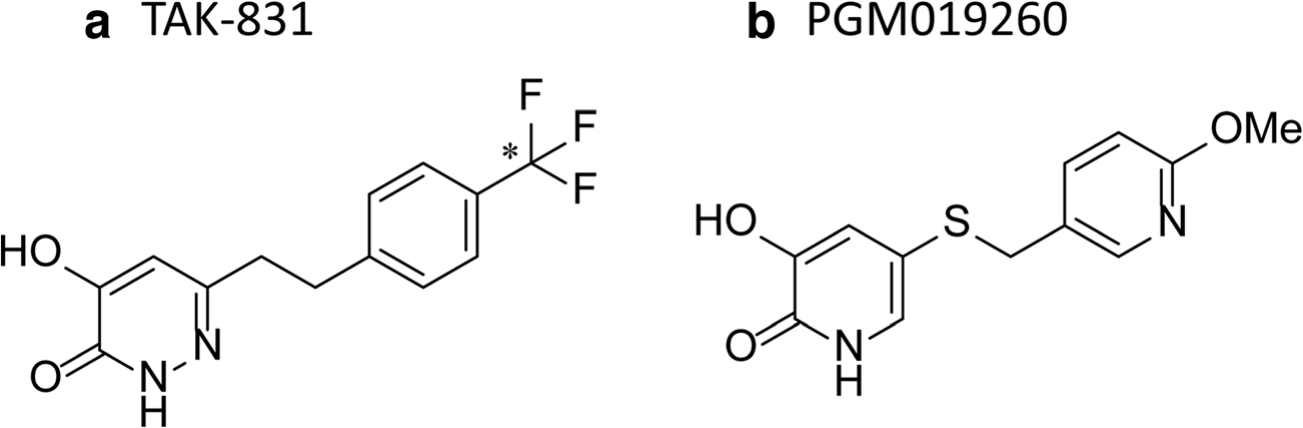
Chemical structures of (**a**) TAK-831 (asterisk donates ^14^C-labeled position) and (**b**) PGM019260.

TAK-831 and PGM019260 were synthesized by Takeda Pharmaceutical Company Limited. [^14^C]TAK-831 (specific activity: 7.46 MBq/mg, radiochemical purity: 97.3%) was synthesized by Curachem, Inc. All other chemicals and reagents were obtained from commercial sources. Chemical structures of [^14^C]TAK-831 and PGM09260 are shown in Fig. 1. PGM019260 was used as a tracer to measure target occupancy of DAAO. PGM019260 has been characterized that 1) PGM019260 showed highly potent DAAO inhibition in human and mouse enzymes in vitro and 2) pre-treatment of sodium benzoate, a DAAO inhibitor, caused a dose dependent displacement of PGM019260 in the mouse cerebellum (6).

### Animals

C57BL/6 J mice (male, 30 ± 10 g, 10–11 weeks) purchased from Cadila (India) were used for target occupancy study. C57BL/6 J mice (male, 23 ± 5 g, 8 weeks) purchased from Charles River (Japan) were used for brain distribution study. All animals were housed under standard laboratory conditions on a 12 h light-dark cycle. Food and water were provided ad libitum. The target occupancy study was performed in compliance to Institutional Animal Ethics Committee under principles of Good Research Practice of Suven Life Science. The pharmacodynamic and brain distribution studies were performed under approval by the Institutional Animal Care and Use Committee at Takeda Pharmaceutical Company. Summary of group allocation of C57BL/6 J mice in preclinical studies of TAK-831 is shown in Supplementary Table S1.

### Experimental Procedure

In the target occupancy study, TAK-831 was formulated in 1% *v*/v Tween 80 and 99% v/v of 0.5% *w*/*v* methyl cellulose. PGM019260 was formulated in 5% dimethyl sulfoxide and 95% v/v of 20% w/v 2-hydroxypropyl-β-cyclodextrin. The vehicle (1% v/v Tween 80 and 99% v/v of 0.5% w/v methyl cellulose) or TAK-831 at 0.3, 1.0, 3.0 and 10 mg/kg were orally administered (p.o.) with a dose volume of 10 mL/kg. The dose levels were aligned with an efficacy study (novel object recognition test) in which efficacy was observed at ≥0.3 mg/kg acute oral dose in mice (7). After 2, 4, 6, 8, 10 and 24 h from oral administration, PGM019260 at 60 μg/kg was administered by intravenous route (i.v.) with a dose volume of 1 mL/kg (*n* = 4). Mice were sacrificed 20 min after tracer dosing. Blood was collected into heparin containing tubes and plasma was separated. Two separate brain regions (cerebellum and frontal cortex) were isolated and frozen on dry ice until further processed for extraction and analysis of TAK-831 or PGM019260 using liquid chromatography-tandem mass spectrometry (LC/MS/MS).

In the pharmacodynamics study, TAK-831 was formulated in 1% *v*/v Tween 80 and 99% v/v of 0.5% *w*/*v* methyl cellulose. TAK-831 at 0.3, 1, 3 and 10 mg/kg was administered p.o. with a dose volume of 10 mL/kg (*n* = 7). After 2, 6, 10 and 24 h of oral administration, mice were sacrificed and cerebellum were isolated and frozen on dry ice until further processed for analysis of D-serine using high performance liquid chromatography (HPLC).

In the brain distribution study, [^14^C]TAK-831 was formulated in 0.5% *w*/*v* methyl cellulose. [^14^C]TAK-831 at 0.3 and 3 mg/kg (specific activity: 7.46 MBq/mg) was administered p.o. with a dose volume of 10 mL/kg. Mice were sacrificed (*n* = 2) 0.25, 0.5, 1, 2, 4, 8 and 24 h after p.o. dosing. Blood was collected with heparinized syringe and plasma was separated. Three separate brain regions (cerebellum, front and back part of cerebrum) were isolated. The radioactivity in plasma and brain regions was measured by liquid scintillation counter. For metabolite profiling purpose, [^14^C]TAK-831 at 3 mg/kg was administered p.o. Mice were sacrificed (*n* = 2) 0.25, 1 and 6 h after p.o. dosing and plasma and whole brain homogenate samples were obtained. Plasma and whole brain homogenate samples were processed for metabolite profiling by HPLC with on-line radioisotope detector or liquid scintillation counter.

### Analysis of TAK-831 and PGM019260

TAK-831 and PGM019260 were quantified by LC/MS/MS. Briefly, TAK-831 in mouse plasma was extracted with acetonitrile. PGM019260 in mouse cerebellum and frontal cortex were homogenized and extracted by acetonitrile. The pretreated samples were analyzed by LC/MS/MS with the analytical column of X Bridge C18 (2.1 × 50 mm, 3.5 μm) at 40°C. The mobile phases were the mixture of 10 mM ammonium formate (0.2% formic acid) and acetonitrile with the flow rate of 0.5 mL/min under gradient program. TAK-831 and PGM019260 were detected by multiple reaction monitoring under positive ion mode with *m/z* transitions 285 → 126 for TAK-831 and 265 → 108 for PGM019260, respectively. TAK-831 was quantified in a calibration range of 2.0–500 ng/ml in plasma. PGM019260 was quantified in a calibration range of 0.1–50 and 0.05–500 ng/g (cerebellum and frontal cortex). Twenty tissue samples of whole brain were collected from untreated mice for blank matrix.

### Analysis of D-Serine

D-serine in mouse cerebellum was quantified by HPLC after derivatization with *ortho*-phthalaldehyde (OPA) and N-*tert*-butyloxycarbonyl-L-cysteine (Boc-L-Cys) (15–17). Briefly, after mouse cerebellar sample was homogenized and extracted by methanol, sodium borate was added. After vortex mixing and centrifugation, the mixture of OPA and Boc-L-Cys was added for derivatization reaction. The prepared sample was analyzed by HPLC with the analytical column of TSKgel ODS-80Ts QA at 40°C. The mobile phases were the mixture of sodium acetate (pH 6) and acetonitrile with the flow rate of 0.25 mL/min under gradient program. Fluorescence detection of the D-serine derivative was carried out at 443 nm with excitation at 344 nm. The calibration range was set at 0.5–200 ng/g in mouse cerebellum.

### Mechanistic Multilayer PK/TO/PD Model Analysis

**Fig. 2:**
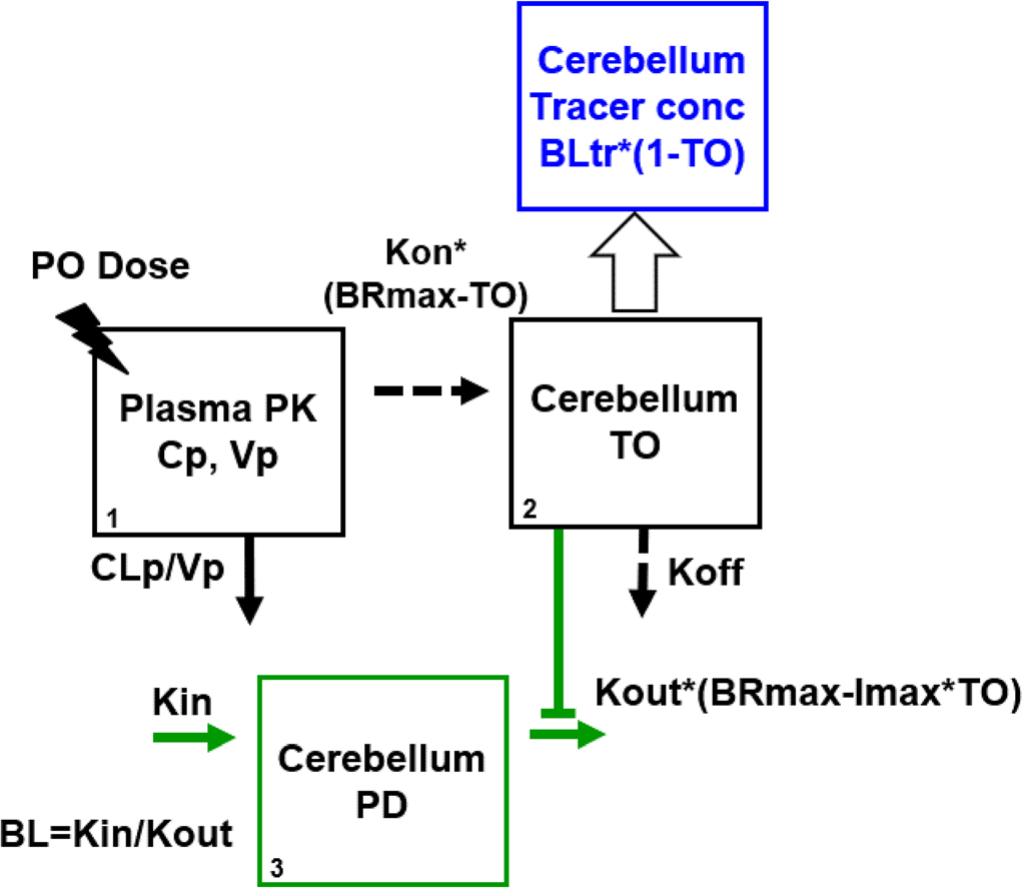
Schematic description of developed mechanistic multilayer model structure of PK/TO/PD of TAK-831 in mice. Plasma PK was described by 1-compartment model with linear elimination and connected with cerebellum TO with mechanistic binding kinetics model to target enzyme, DAAO. TO of TAK-831 was measured as the magnitude of tracer replacement. The PD modulation was described by indirect response model with inhibition of PD elimination dependent on TO. Where Cp, Vp and CLp represent concentration, oral clearance and apparent distribution volume of TAK-831 in plasma PK compartment, respectively. Kon, Koff and BRmax represent second-order association rate constant, first-order dissociation rate constant and maximum binding ratio of TAK-831 in cerebellum TO compartment, respectively. BLtr represents baseline concentration of tracer in cerebellum. Kin, Kout, BL represent zero-order generation rate constant, first-order elimination rate constant and baseline concentration of D-serine in cerebellum PD compartment, respectively. Imax represents maximum inhibitory effect of TO on Kout.

Figure 2 depicts the schematic description of the developed PK/TO/PD model. The PK model consists of a one compartment model with a first-order elimination. Since the absorption phase was not clearly observed, TAK-831 dosage was dealt with as a direct injection into plasma PK compartment. The PK/TO relationship of TAK-831 was described by mechanistic binding kinetics model (18), which accounts for the association and dissociation rates to the target enzyme in relation to the plasma concentration of TAK-831 assuming a rapid equilibrium between plasma and brain free concentrations. Additional simulation was performed utilizing estimated unbound cerebellum concentrations of TAK-831 as PK input for TO model analysis to support this assumption (Supplementary information). Mass transfer of TAK-831 due to association to and dissociation from the target enzyme was not considered between plasma and cerebellum compartments since the amount of target enzyme is limited in cerebellum. The PK/TO model analysis was performed against specifically-bound tracer, PGM019260, concentration in cerebellum, which was calculated by subtracting tracer concentration in frontal cortex (reference region) from that in cerebellum (target region). The grand mean value of tracer concentration in vehicle treatment group calculated from all the time points (2, 4, 6, 8, 10 and 24 h after vehicle treatment) was used as the baseline of tracer concentration across all the time points. The PK/TO model was further connected with D-serine concentration time-profiles in cerebellum by indirect response model where D-serine elimination was inhibited depending on the magnitude of TO by TAK-831. The differential equations of PK/TO/PD model are shown below (Eqs. 1, 2 and 5). TO of TAK-831 was converted to tracer concentration by Eq. 3. In vivo dissociation constant (Kd) and zero-order generation rate constant of D-serine (Kin) were calculated by Eqs. 4 and 6, respectively.

1 $$ \frac{dAp}{dt}=-\frac{CLp}{Vp}\cdotp Ap $$

2 $$ \frac{dTO}{dt}= Kon\cdotp \left( BRmax- TO\right)\cdotp Cp- Koff\cdotp TO $$

3 $$ \mathrm{Tracer}\ \mathrm{concentration}= BLtr\cdotp \left(1- TO\right) $$

4 $$ In\ vivo\  Kd=\frac{Koff}{Kon}\times fp $$

5 $$ \frac{dPD}{dt}= Kin- Kout\cdotp \left( BRmax- Imax\cdotp TO\right) $$

6 $$ Kin= BL\cdotp Kout $$

Where Ap, Cp, CLp and Vp represent amount, concentration, oral clearance and apparent distribution volume of TAK-831 in plasma PK compartment, respectively. Cp is calculated by Ap/Vp. TO, Kon, Koff, Kd and BRmax represent target occupancy, second-order association rate constant, first-order dissociation rate constant, equilibrium dissociation constant and maximum binding ratio of TAK-831 in cerebellum TO compartment, respectively. BLtr represents baseline tracer concentration in cerebellum. PD, Kin, Kout, BL represent concentration, zero-order generation rate constant, first-order elimination rate constant and baseline concentration of D-serine in cerebellum PD compartment, respectively. Imax represents maximum inhibitory effect of TO on Kout. fp represents the free fraction of TAK-831 in mouse plasma (0.077).

The PK/TO/PD model analysis was performed using NONMEM VI (ICON Development Solutions) by means of First-Order Conditional Estimation method with Interaction (FOCE). The convergence criterion was three significant digits. A Compaq Digital Fortran Version 6.1 compiler (Compaq Computer Corporation) was used to compile and execute NONMEM. All the analyses were conducted by naive pool method and inter-individual variability in the parameters was not estimated in the present analyses. The proportional error model was used for all PK, TO and PD readouts and experimental observations below the limit of quantification was excluded from the analysis. Model selection was based on the visual inspection of goodness-of-fit, the precision of parameter estimates and the minimum value of the objective function. The simulation based on the established PK/TO/PD model was performed using Berkeley Madonna, version 8.3.14 (University of California).

## Results

### Empirical Data Collection of Plasma PK, Cerebellum TO and Cerebellum PD in Mice

**Fig. 3:**
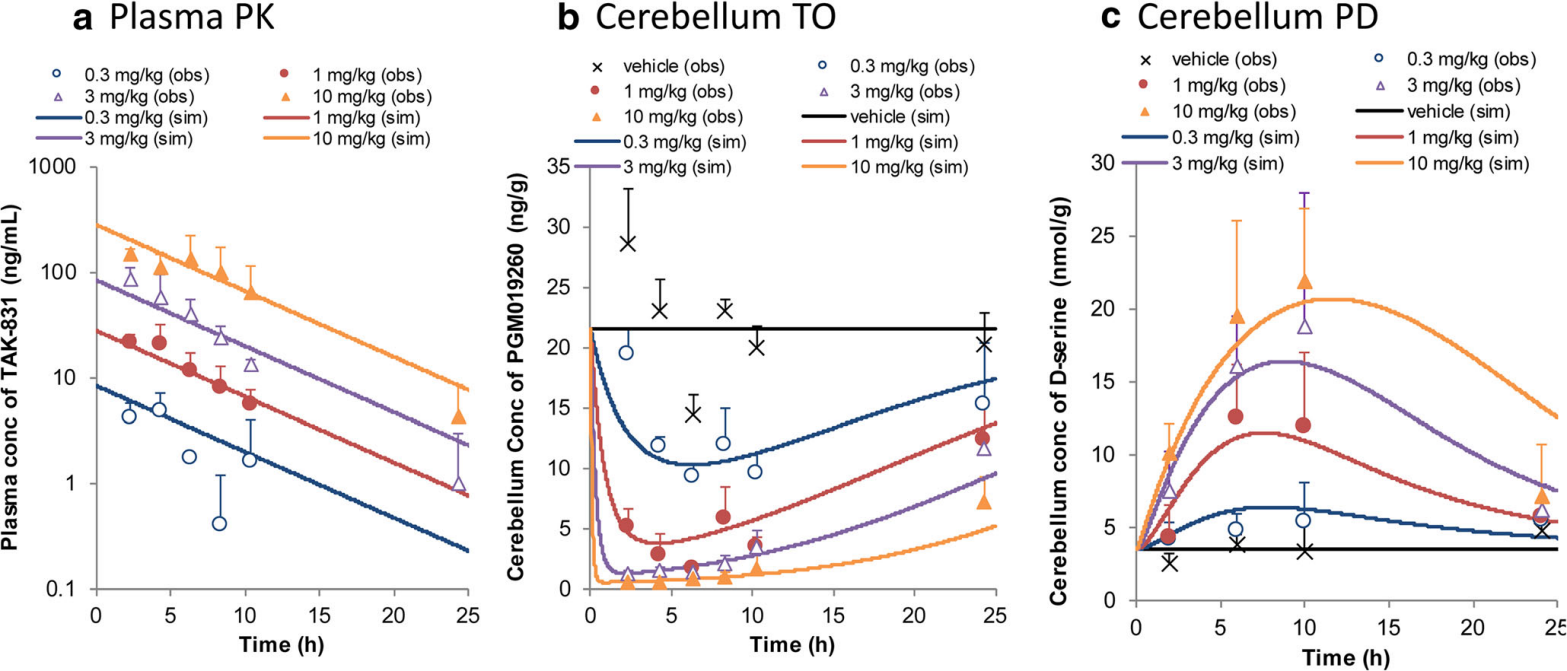
The empirical observations and mechanistic multilayer model analysis on PK/TO/PD relationship after a single oral administration of TAK-831 in mice. Observed and model simulated (**a**) mean plasma concentration-time profiles of TAK-831 and (**b**) mean concentration-time profiles of the specific binding of PGM019260 in cerebellum after a single oral administration of TAK-831 at 0.3, 1, 3 and 10 mg/kg followed by a single intravenous administration of PGM019260 at 20 min before blood and brain sampling in mice (*n* = 4). (**c**) Observed and model simulated mean concentration-time profiles of D-serine in cerebellum after a single oral administration of TAK-831 at 0.3, 1, 3 and 10 mg/kg in mice (*n* = 7). In each panel, symbols, error bars and lines represent the mean observed data, standard deviations and the model simulations, respectively. Obs, observed; sim, simulated.

Mean concentration-time profiles of TAK-831 in plasma and the specific binding of PGM019260 in cerebellum after a single oral administration of vehicle or TAK-831 followed by a single intravenous administration of PGM019260 at 20 min before sampling are shown in Fig. 3a and b, respectively. The plasma exposure of TAK-831 increased almost dose-proportionally within dose range from 0.3 to 10 mg/kg. The absorption phase was not clearly observed since first sampling time point was 2 h after oral administration of TAK-831. The tracer, PGM019260, concentration in cerebellum decreased with increasing dose of TAK-831. The maximum decline of tracer concentration was observed with TAK-831 dosage at 3 mg/kg or above. An obvious time-lag was observed between plasma PK and cerebellum TO, where Tmax of plasma PK was 2 h or earlier while that of TO was at 4 or 6 h, especially at low dose of TAK-831. The possible causality was deemed the slow brain penetration through blood brain barrier (BBB) and/or slow binding kinetics to target enzyme although these two factors could not be distinguished in this target occupancy experiment. Additionally, the relatively large variability was observed in tracer concentrations especially in vehicle dosing group. The concentration-time profile of D-serine in cerebellum is shown in Fig. 3c. D-serine level in cerebellum increased dose-dependently.

### Brain Distribution of [^14^C]TAK-831 in Mice

**Fig. 4:**
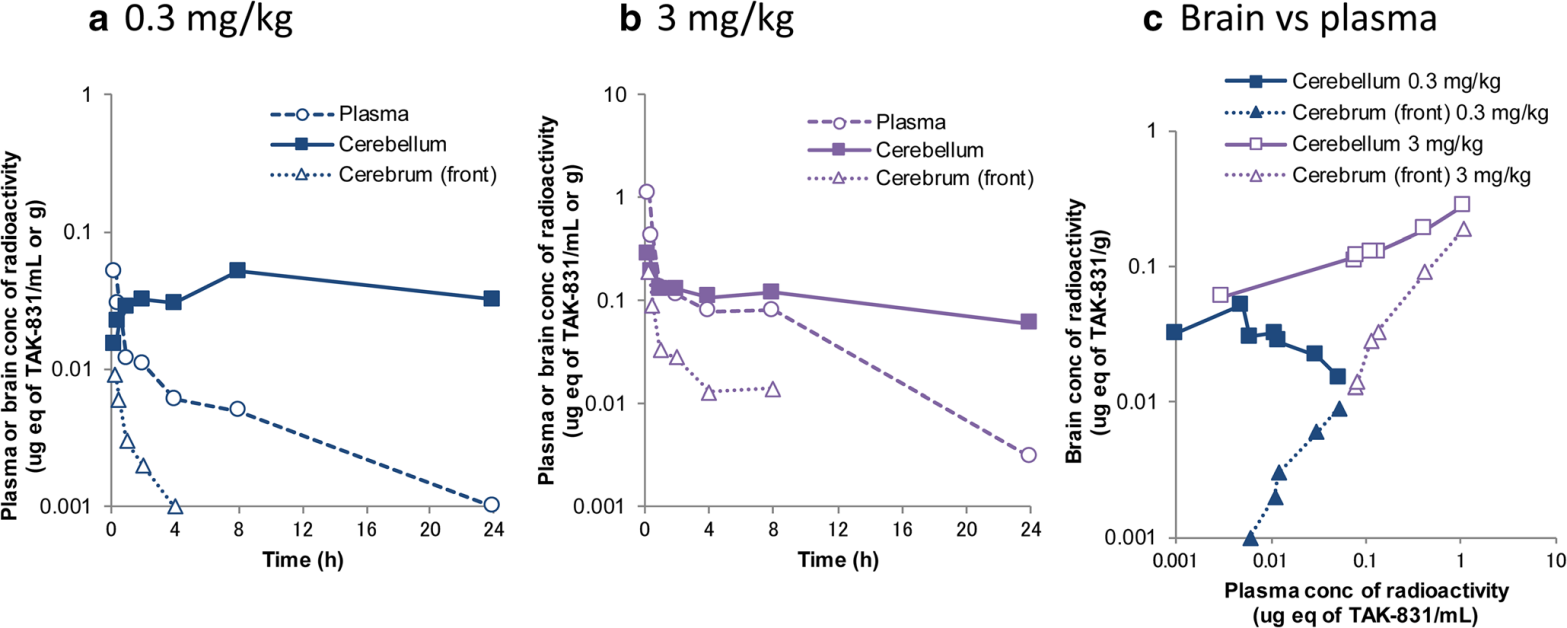
Concentration- and time-dependent brain distribution of TAK-831 in mice. Mean plasma concentration-time profiles of radioactivity in plasma, cerebellum (target region) and front part of cerebrum (reference region) at (**a**) 0.3 mg/kg and (**b**) 3 mg/kg, and (**c**) their correlation between cerebellum or front part of cerebrum and plasma after a single oral administration of [^14^C]TAK-831 at 0.3 and 3 mg/kg in mice (*n* = 2). In each panel, symbols represent the mean observed data (*n* = 2).

Since time-lag was observed between plasma PK and cerebellum TO, brain distribution of [^14^C]TAK-831 was investigated. The mean concentration-time profiles of radioactivity in plasma, cerebellum (target region) and front of cerebrum (reference region) are shown in Fig. 4a and b. Plasma concentration of radioactivity reached Cmax at 0.25 h followed by bi-phasic elimination. The plasma exposure of radioactivity increased almost dose-proportionally from 0.3 to 3 mg/kg. On the contrary, an obvious difference was observed in the time profiles of radioactivity in cerebellum between 0.3 and 3 mg/kg. Metabolite profiling was performed for plasma and whole brain homogenate at 0.25, 1 and 6 h after a single oral administration of [^14^C]TAK-831. The percentage of TAK-831 to total radioactivity were from 61.4% to 76.0% in plasma and from 88.0% to 97.5% in brain, respectively. These results indicated that the main component in both plasma and brain was unchanged TAK-831 in mice and there was no obvious time-dependent change in the proportion of TAK-831 in either plasma or brain. The correlation plot between brain (cerebellum or front part of cerebrum) and plasma concentration of radioactivity is shown in Fig. 4c. A good correlation was observed between cerebrum and plasma throughout wide concentration range and time period, suggesting that TAK-831 rapidly penetrated BBB and reached the equilibrium between cerebrum and plasma. On the other hand, the correlation plot between cerebellum and plasma was quite different especially at low dose level (0.3 mg/kg) where an obvious anti-clockwise hysteresis was observed. At high dose at early time points, the correlation between cerebellum and plasma was close to that between cerebrum and plasma, suggesting that the specific binding was saturated and non-specific binding became the main component in cerebellum. From these results combined with high and specific expression of DAAO in cerebellum (1), it was suggested that observed distinct target occupancy-time profiles was mainly caused by the interaction with target enzyme, DAAO, rather than slow penetration of BBB.

### Mechanistic Multilayer PK/TO/PD Model Analysis and Simulation

The quantitative relationship among PK, TO and PD in mice was parsimoniously connected by the PK/TO/PD model schematically described in Fig. 2. The sequential approach was taken in the multilayer modeling analysis; at first, PK model analysis was solely carried out and the estimated PK model parameters were fixed in the subsequent PK/TO model analysis. Likewise, the PD components was analyzed after PK/TO model parameters were fixed. The overlay of model-simulated and observed plasma concentration of TAK-831 (Fig. 3a), tracer concentration in cerebellum (Fig. 3b) and D-serine concentration in cerebellum (Fig. 3c) are shown, respectively. The estimated model parameters and residual errors are summarized in Table I. All the parameters were estimated with good precision.

**Table I:** The Tabulated Model Parameters for PK/TO/PD of TAK-831 in Mice

Category	Parameter	Unit	Definition	Value	RSE (%)
PK	CLp	L/h/kg	oral clearance	5.14	6.07
	Vp	L/kg	apparent volume of distribution	35.7	9.75
	residual error	%	proportional residual error	54.0	15.2
TO	Kon	mL/ng/h	association rate constant	0.0372	8.68
	Koff	1/h	dissociation rate constant	0.113	13.1
	BRmax	–	maximum binding ratio	0.987	0.579
	BLtr	ng/g	baseline concentration of tracer	21.6	Fix
	residual error	%	proportional residual error	41.6	22.4
PD	BL	nmol/g	baseline concentration of D-serine	3.48	5.06
	Kout	1/h	elimination rate constant	1.32	11.3
	Imax	–	maximum inhibitory effect	0.877	2.42
	residual error	%	proportional residual error	34.4	15.8

-: not applicable; RSE, relative standard error; PK, pharmacokinetics; TO, target occupancy; PD, pharmacodynamics

For PK/TO relationship, three models were evaluated. The equations used in addition to final model are provided as Supplementary information. First, direct response model was tested assuming that plasma concentration and TO in cerebellum rapidly reaches the equilibrium. As it was presumed from the observed time-lag between plasma PK and cerebellum TO, the model could not well describe the tracer concentrations at low dose groups, especially at earlier time points. In order to address this time-lag, the effect compartment-Emax model and mechanistic binding kinetics model were tested. While both models significantly improved the goodness-of-fit compared with the direct response model (decrease in objective function: 17.6 and 53.5, respectively), the mechanistic binding kinetics model achieved significantly better fitting results as this model could capture the dose-dependent time-lag at low dose groups. The PK/TO model developed by mechanistic binding kinetics model captured the concentration- and time-dependent PK/TO relationship within wide dose range, except for the mismatch observed at 2 h at 0.3 mg/kg group. This mismatch may have been caused by the observed large variability in vehicle treatment group. The inclusion of maximum binding ratio (BRmax, estimated value: 0.987) significantly improved the goodness-of-fit (decrease in objective function: 11.4), possibly representing the small amount (~1.3%) of non-specifically bound tracer, which could not be replaced by high concentration of TAK-831. The established PK/TO model was further connected with PD, D-serine in cerebellum. Indirect response model with inhibition of D-serine elimination (Kout) was employed according to the mechanism of action of TAK-831. The developed PK/TO/PD model well described the PD time profiles in the wide dose range. D-serine concentrations at 24 h were slightly over-predicted at high dose levels. The potential reasons could be the slight over-prediction of TO and/or inter-study variability.

**Fig. 5:**
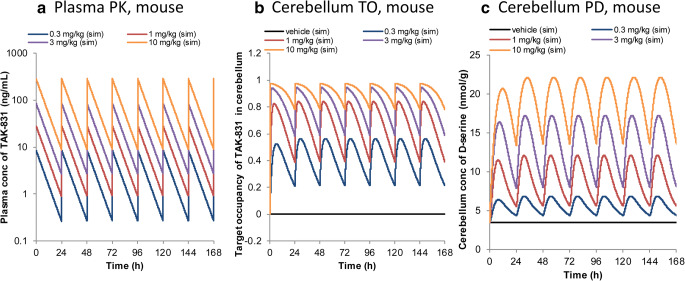
The established mechanistic multilayer PK/TO/PD model-based simulation of (**a**) plasma concentration of TAK-831, (**b**) target occupancy of TAK-831 in cerebellum and (**c**) D-serine concentration in cerebellum after multiple oral administrations of TAK-831 at 0.3, 1, 3 and 10 mg/kg once daily in mice.

The multiple dose simulation was performed using the established PK/TO/PD model. The simulated PK in plasma, TO and D-serine concentration profiles in cerebellum after multiple oral administrations of TAK-831 at 0.3, 1, 3 and 10 mg/kg once daily in mice are shown in Fig. 5. The simulation results indicated that TO and PD in addition to PK reached the steady state within a few days and there was no obvious accumulation of TO or PD after multiple oral administration of TAK-831 under once daily dose schedule in mice. The simulated results were in good agreement with the empirical observations in the separate studies, where no accumulation was observed for PK or TO after once daily multiple doses of TAK-831 in mice (data not shown).

## Discussion

In discrete mouse PK study with more frequent blood sampling including early time points, plasma PK of TAK-831 showed rapid absorption followed by bi-phasic elimination, which was quite similar to that observed after [^14^C]TAK-831 administration as shown in Fig. 4a and b. The 2-compartment PK model was developed for those discrete mouse PK and used as PK input for PK/TO model analysis. The analysis showed no obvious difference observed in the estimated PK/TO model parameters and fitting results (data not shown), suggesting that the absorption and alpha phases of TAK-831 PK did not have a large impact on the PK/TO model analysis performed in this study. The large variability was observed in tracer, PGM019260, profiles in cerebellum especially after vehicle dosing. Since no information is available on the circadian rhythm of DAAO expression to the best knowledge of the authors and there was no obvious variability observed in the baseline D-serine concentrations (Fig. 3c), the grand mean concentration of PGM019260 in vehicle group was used as the overall tracer baseline in the subsequent model analysis. To access the impact of fluctuating baseline tracer concentrations on subsequent TO model analysis, the separate data analysis was performed by normalizing the tracer concentrations in TAK-831 coadministration groups at each time point to extract the change from the baseline. As the result, no major difference was found between observed and normalized PGM01926 profiles (data not shown), supporting the use of grand mean concentration of PGM019260 in vehicle group as the baseline for subsequent TO model analysis. Nevertheless, it would be a good idea to measure the plasma concentration of PGM019260, which was not measured in the present study, from the same individual animal to address the observed baseline tracer variability. The nonlinear mixed-effect modeling approach may be able to compensate the observed inter-individual variability with taking the individual relationship between plasma and brain concentrations into account.

The PK/PD model analysis of DAAO inhibitors has been previously performed by Strick et.al. (4). In their study, the relationship between free brain concentration of DAAO inhibitors and D-serine concentration in cerebellum was analyzed in mice. In our PK/TO model analysis, the plasma total concentrations of TAK-831 was used as a surrogate of unbound cerebellum concentrations from following reasons: 1) BBB permeability of TAK-831 was rapid and quickly reached an equilibrium by 0.25 h in mice (Fig. 4c); 2) unbound brain-to-plasma ratio (Kp,uu) of TAK-831 was considered to be 1, since TAK-831 is neither a substrate of P-glycoprotein (P-gp) nor breast cancer resistance protein (BCRP). This assumption is supported by recent literatures suggesting that P-gp and BCRP are the two most abundant BBB efflux transporters in human, nonhuman primate, mouse and rat (19–22); 3) it is generally considered the rate-limiting step of whole equilibrium process including plasma protein binding, BBB transport and brain non-specific binding is often BBB transport (23). Under these assumptions, additional simulation was performed utilizing estimated unbound cerebellum concentrations of TAK-831 as PK input for TO model analysis (Supplementary information). The simulated TO time-profiles indicated that cerebellum unbound concentration-based TO estimation was quite similar to that estimated by plasma total concentrations as PK input (Supplementary Fig. S1(c)), supporting the use of plasma concentrations of TAK-831 as the surrogate of unbound concentration in cerebellum. On the contrary, if slow BBB penetration was assumed, the rapid equilibrium observed in Fig. 4c could not be reproduced, confirming that observed distinct target occupancy-time profiles was mainly caused by the interaction with DAAO enzyme.

The indirect response model with inhibition of D-serine elimination was commonly used by both Strick et al. and our group. The system related parameters were quite comparable between Strick et.al. (4) and our study. The estimated D-serine elimination rate constants (Kout) were 2.10 and 1.32 1/h, respectively. The maximum inhibitory effect of D-serine elimination (Imax) were 0.843 and 0.877, respectively, suggesting that DAAO is the enzyme mainly responsible for D-serine elimination while a small portion of elimination activity was left even under the full occupation of DAAO. The in vivo equilibrium dissociation constant (Kd) calculated by Eq. 4 in our study (0.23 ng/mL) was about 8-fold lower than the 50% inhibitory concentration (IC50) of DAAO activity in vitro (1.8 ng/mL). Interestingly, the observed gap between in vivo and in vitro was also consistent with Strick et.al. where the in vivo IC50 was about 20-fold lower than in vitro IC50. Although the definitive reason behind this gap is unclear, similar findings have been frequently reported with possible explanations: 1) hypothesis that not unbound but total brain concentration playing a role (24), 2) not at equilibrium state in vivo or in vitro (24–26) and 3) internalization/down regulation of target in vivo preventing the tracer from binding (14). In addition to in vitro to in vivo difference in equilibrium binding affinity/potency, the kinetic difference was observed between plasma PK and cerebellum TO of TAK-831, which was not observed for a structure analogue of TAK-831. Neither in vivo brain TO nor whole brain PK could have distinguished the possible mechanism driving the observed kinetic difference: slow BBB penetration, slow target-mediated binding kinetics or their combination. From these observations, it should be emphasized that in vivo evaluation of TO time profiles is beneficial to characterize not only more relevant in vivo equilibrium binding affinity/potency but also its accompanying kinetics. In combined with tissue distribution to target and reference regions, the empirical and modeling framework performed in this study would provide a quantitative means to fully elucidate the quantitative, mechanistic and kinetic relationship across pharmacokinetics, target engagement and subsequent modulation of biopathological pathway of interest.

In our preclinical studies, social interaction study was performed in Balb/c mice while PK/TO/PD relationship was examined in C57BL/6 mice. Although BBB penetration of TAK-831 was not examined in Balb/c mice, the brain exposure of unbound TAK-831 would not be remarkably different from C57BL/6 mice. This is based on the literature information that the BBB permeability of sodium fluorescein, a marker of BBB permeability, was similar or slightly higher in Balb/c mice than C57BL/6 mice (27), combined with the fact that TAK-831 showed rapid BBB penetration in C57BL/6 mice (Fig. 4c). Similarly, it is suggested that the brain exposure of unbound TAK-831 in schizophrenia patients may not be remarkably different from that in healthy subjects since the BBB permeability is reported to be increased in schizophrenia patients (28).

The established PK/TO/PD model in mice was further translated to humans in order to design a PET imaging study for the investigation of TAK-831 TO in humans and to estimate associated change in D-serine. The mechanistic insights revealed in preclinical studies served as an important basis for the model translation to humans. Model translation steps are detailed in Supplementary information. Briefly, 1) PK model was replaced by the human PK model predicted from rats with single species allometric scaling approach (29); 2) TO model accounted for the species difference in plasma protein binding on association rate constant (Kon); 3) PD model considered the possible species difference in D-serine turnover in cerebellum, which was estimated from the plasma half-lives of D-serine between mice (t1/2,mouse: 1.2 h) (30) and schizophrenia patients (t1/2,human: 3.3 h) (31). The translated model-simulated PK, TO, PD profiles in humans are shown in Supplementary Fig. S2. The model parameters used for simulation are summarized in Supplementary Table S2. The translated PK/TO/PD model was especially useful to quantitatively select the dose level and PET scanning time points; one is at a little later than time at maximum plasma concentration of TAK-831 and another is after 24 h with consideration of the time-lag between the plasma PK and cerebellum TO simulated in humans.

D-serine concentration in cerebellum measured and modeled in this study is not a measurable biomarker in humans. Due to technical difficulties with quantitative measurement of D-serine in cerebrospinal fluid (CSF) in mice, the quantitative relationship of D-serine between cerebellum, CSF and plasma was investigated in rats in a separate study (manuscript under preparation). The understanding of the quantitative relationship in biomarker change between target organ and clinically accessible biological matrices is of crucial importance to demonstrate the magnitude of clinical proof of mechanism. Since mouse was the primary pharmacological model species, the cerebellum D-serine response around efficacious dose levels evaluated and modeled in this study would be mathematically integrated with the quantitative relationship with CSF and plasma. With emerging clinical PK, TO, PD information after treatment of TAK-831, the developed quantitative modeling framework should be further translationally calibrated to be a more robust quantitative means for pursuing model-informed biomarker based clinical drug development.

## Conclusion

In this study, a quantitative multilayer mechanistic model which describe the nonlinear PK/TO/PD relationship of TAK-831 was established in mice. Since discrete kinetics were observed between plasma PK and cerebellum TO in mice, the brain distribution of TAK-831 in cerebellum (target region) and front part of cerebrum (reference region) was investigated. The results revealed that target-mediated slow binding kinetics, rather than slow penetration of BBB, would mainly contribute to the observed discrete target engagement kinetics in cerebellum. The PK/TO/PD relationship was parsimoniously connected with the mechanistic binding kinetics model and indirect response model, respectively. The developed model well characterized the concentration- and time-dependent nonlinear PK/TO/PD relationship after an oral administration of TAK-831 in mice.

The established model was useful to quantitatively understand the nonlinear PK/TO/PD relationship and their dose-dependency around the efficacious dose in mice. Moreover, the model was translationally utilized to simulate PK/TO/PD relationship in humans, which supported the study design of clinical PET study for optimal dose and PET scanning time point to investigate the target engagement in humans. In conclusion, the established empirical and mechanistic modeling framework would provide a quantitative and translational means in the multilayer biomarker-assisted drug discovery and development in multiple CNS indications.

## Electronic supplementary material

ESM 1 (DOCX 94.6 kb)

ESM 2 (DOCX 77 kb)

ESM 3 (DOCX 76 kb)

ESM 4 (PNG 580 kb)

High Resolution Image (TIF 4186 kb)

ESM 5 (PNG 658 kb)

High Resolution Image (TIF 4164 kb)

## Abbreviations

BBB: Blood brain barrier
BCRP: Breast cancer resistance protein
Boc-L-Cys: N-*tert*-butyloxycarbonyl-L-cysteine
CIAS: Cognitive impairment associated with schizophrenia
CNS: Central nervous system
CSF: Cerebrospinal fluid
DAAO: D-amino acid oxidase
FOCE: First-order conditional estimation
GluRδ2: Delta 2 glutamate receptor
HPLC: High performance liquid chromatography
IC50: 50% inhibitory concentration
i.v.: Intravenously
LC/MS/MS: Liquid chromatography-tandem mass spectrometry
NMDA: *N*-methyl-*D*-aspartate
OPA: *ortho*-phthalaldehyde
PET: positron emission tomography
P-gp: P-glycoprotein
p.o.: per orally
PK: Pharmacokinetics
PD: Pharmacodynamics
TO: Target occupancy
