# Supplementary material for: Mechanistic Multilayer Quantitative Model for Nonlinear Pharmacokinetics, Target Occupancy and Pharmacodynamics (PK/TO/PD) Relationship of D-Amino Acid Oxidase Inhibitor, TAK-831 in Mice
Source: Pharm Res. 2020 Aug 5;37(8):164. doi: 10.1007/s11095-020-02893-x (PMC7478952; doi:10.1007/s11095-020-02893-x)
Supplement: Supplementary file 1 — (DOCX 94.6 kb) [file 11095_2020_2893_MOESM1_ESM.docx]

# Supplementary information

PK/TO model analysis

For PK/TO relationship, other two models, the direct model and the effect compartment-Emax model, were evaluated. In the direct model, plasma PK and TO were calculated by Eq.1 and Eq.S1, respectively. In effect compartment-Emax model, plasma PK and PK in effect compartment were calculated by Eq.1 and Eq.S2, and TO was calculated by Eq.S3.

$$\mathrm{TO}=\frac{BRmax\cdot Cp}{Kd+Cp} (Eq. S1)$$

$$\frac{dAe}{dt}=ke0\cdot(Ap-Ae) (Eq.S2)$$

$$\mathrm{TO}=\frac{BRmax\cdot Ce}{Kd+Ce} (Eq. S3)$$

Where Ae and Ce represent amount and concentration of TAK-831 in effect compartment, respectively. Ce is calculated by Ae/Vp. Ke0 and Kd represent target occupancy, first-order rate constant to/from effect compartment and dissociation rate constant, respectively.

Comparison between plasma total concentrations and estimated unbound cerebellum concentrations of TAK-831 as input for TO model analysis

In our PK/TO model analysis, the plasma total concentrations of TAK-831 was used as a surrogate of unbound cerebellum concentrations from following reasons: 1) BBB permeability of TAK-831 was rapid and quickly reached an equilibrium by as early as 0.25 h in mice as indicated in Figure 4(C); 2) unbound brain-to-plasma ratio (Kp,uu) of TAK-831 was considered to be 1, since TAK-831 is neither a substrate of P-glycoprotein (P-gp) nor breast cancer resistance protein (BCRP). This assumption is supported by recent literatures suggesting that P-gp and BCRP are the two most abundant BBB efflux transporters based on proteomics approach quantifying transporter protein expression in brain microvessel preparations from human, nonhuman primate, mouse and rat([1-4](#_ENREF_1)); 3) it is generally considered the rate-limiting step of whole equilibrium process including plasma protein binding, BBB transport and brain non-specific binding is often BBB transport([5](#_ENREF_5)).

Under these assumptions, additional simulation was performed utilizing estimated unbound cerebellum concentrations of TAK-831 as PK input for TO model analysis. The unbound cerebellum concentrations of TAK-831 was calculated by following steps: 1) cerebrum-to-plasma concentration ratio (Kp_cerebrum_: 0.181) of TAK-831 was estimated from a good correlation of TAK-831 concentrations between cerebrum and plasma (Supplementary figure S1(A)); 2) total cerebrum concentration of TAK-831 (C_cerebrum_) was calculated with Eq. S4; 3) free fraction of TAK-831 in cerebrum (f_cerebrum_: 0.425) was calculated by Eq. S5 assuming that Kp,uu equals 1; 4) unbound plasma (Cp_,unbound_) and cerebrum (C_cerebrum,unbound_) concentrations of TAK-831 were calculated by Eq S6 and S7, respectively; 5) unbound cerebellum concentrations of TAK-831 (C_cerebellum,unbound_) were considered equal to that in cerebrum (Eq. S7).

$$C_{cerebrum}=Cp\cdot{Kp}_{cerebrum} \left( Eq. S4 \right)$$

$$f_{cerebrum}=f_{p}\cdot{Kp}_{cerebrum} (Eq. S5)$$

$${Cp}_{,unbound}=f_{p}\cdot Cp \left( Eq. S6 \right)$$

$$C_{cerebrum, unbound}=f_{cerebrum}\cdot C_{cerebrum}=C_{cerebellum,unbound} (Eq. S7)$$

The model simulated total and unbound concentrations of TAK-831 in plasma and cerebrum after single oral administration of TAK-831 at 3 mg/kg are shown in Supplementary figure S1(B). Under these assumptions, unbound cerebellum concentrations of TAK-831 were quite similar to those in plasma.

In the final step, TO model analysis was performed utilizing estimated unbound cerebellum concentrations of TAK-831 as PK input. The association rate constant, Kon, for unbound cerebellum concentrations of TAK-831 (Kon_cerebellum-unbound_) was calculated by Eq. S8 and incorporated into the modified differential equation (Eq. S9).

$${{Kon}_{cerebellum-unbound}=Kon}_{plasma-unbound}=\frac{{Kon}_{plasma-total}}{fp} (Eq. S8)$$

$$\frac{dTO}{dt}={Kon}_{cerebellum-unbound}\cdot\left( BRmax-TO \right)\cdot C_{cerebellum,unbound}-Koff\cdot TO (Eq. S9)$$

Where Kon_cerebellum-unbound_, Kon_plasma-unbound_, Kon_plasma-total_ represent Kon for unbound cerebellum, unbound plasma and total plasma concentrations of TAK-831, respectively.

PK/TO model analysis was performed utilizing unbound cerebellum concentrations of TAK-831 as PK input, and compared with that utilizing total plasma concentrations of TAK-831 as PK input in Supplementary figure S1(C). The simulated TO time-profiles indicated that cerebellum unbound concentration-based TO estimation was quite similar to that estimated by plasma total concentrations as PK input, supporting the use of plasma concentrations of TAK-831 as surrogate of unbound concentration in cerebellum. On the contrary, if slow BBB penetration was assumed, the rapid equilibrium observed in Figure 4(C) could not be reproduced, confirming that observed distinct target occupancy-time profiles was mainly caused by the interaction with DAAO enzyme.

Translation of PK/TO/PD model to humans

The translational modeling steps from the mouse PK/TO/PD model to that for humans are as follows; 1) PK model was replaced by the human PK model predicted from rats with single species allometric scaling approach([6](#_ENREF_6)). The exponential coefficient of clearance and inter-compartmental clearance was 0.75 and that for central and peripheral volume of distribution was 1, respectively; 2) TO model accounted for the species difference in plasma protein binding between mice (fp,mouse: 0.077) and humans (fp,human: 0.0067), where the apparent association rate constant (Kon) in humans was calculated by Eq. S10. No other modification was made since in vitro IC50 values on enzymatic activity were comparable between mice and humans; 3) PD model considered the possible species difference in D-serine turnover in cerebellum. Since D-serine half-life in human cerebellum could not be found with the best knowledge of authors, we accounted for the species difference in plasma half-lives of D-serine between mice (t1/2,mouse: 1.2 h)([7](#_ENREF_7)) and schizophrenia patients (t1/2,human: 3.3 h)([8](#_ENREF_8)). Elimination rate constant of D-serine (Kout) in human cerebellum was calculated by Eq. S11. The differential equations of translated human PK/TO/PD model are shown in Eq. S12-S14, 2 and 5. Zero-order generation rate constant of D-serine (Kin) were calculated by Eq. 6. The simulated PK, TO, PD profiles in humans after once daily multiple oral administrations of TAK-831 are shown in Supplementary figure S2. The model parameters used for simulation are summarized in Supplementary table 2. With emerging clinical PK, TO, PD information after treatment of TAK-831, the developed quantitative modeling framework should be further translationally calibrated to be a more robust quantitative means for pursuing model-informed biomarker based clinical drug development.

$${Kon}_{human}={Kon}_{mouse}\cdot\frac{{fp}_{human}}{{fp}_{mouse}} (Eq. S10)$$

$${Kout}_{human}={Kout}_{mouse}\cdot\frac{t_{1/2,mosue}}{t_{1/2,human}} (Eq. S11)$$

$$\frac{dX}{dt}=-Ka\cdot X (Eq. S12)$$

$$\frac{dA1}{dt}=F\cdot Ka\cdot-\frac{CL}{V1}\cdot A1-\frac{Q}{V1}\cdot A1+\frac{Q}{V2}\cdot A2 (Eq. S13)$$

$$\frac{dA2}{dt}=\frac{Q}{V1}\cdot A1-\frac{Q}{V2}\cdot A2 (Eq. S14)$$

$$\frac{dTO}{dt}=Kon\cdot\left( BRmax-TO \right)\cdot C1-Koff\cdot TO (Eq. 2)$$

$$\frac{dPD}{dt}=Kin-Kout\cdot(BRmax-Imax\cdot TO)\cdot PD (Eq. 5)$$

$$Kin=BL\cdot Kout (Eq.6)$$

Where X, A1 and A2 represent amount of TAK-831 in absorption, central and peripheral compartment. C1, CL, V1, V2, Q, Ka and F represent concentration in central compartment, clearance, distribution volume of central and peripheral compartments, inter-compartmental clearance, absorption rate constant and bioavailability of TAK-831, respectively. C1 is calculated by A1/V1. TO, Kon, Koff and BRmax represent target occupancy, second-order association rate constant, first-order dissociation rate constant and maximum binding ratio of TAK-831 in cerebellum TO compartment, respectively. PD, Kin, Kout, BL represent concentration, zero-order generation rate constant, first-order elimination rate constant and baseline concentration of D-serine in cerebellum PD compartment, respectively. Imax represents maximum inhibitory effect of TO on Kout.

References

1. Feng B, Doran AC, Di L, West MA, Osgood SM, Mancuso JY, Shaffer CL, Tremaine L, Liras J. Prediction of Human Brain Penetration of P-glycoprotein and Breast Cancer Resistance Protein Substrates Using In Vitro Transporter Studies and Animal Models. J Pharm Sci. 2018;107(8):2225-2235.

2. Uchida Y, Ohtsuki S, Katsukura Y, Ikeda C, Suzuki T, Kamiie J, Terasaki T. Quantitative targeted absolute proteomics of human blood-brain barrier transporters and receptors. J Neurochem. 2011;117(2):333-345.

3. Ito K, Uchida Y, Ohtsuki S, Aizawa S, Kawakami H, Katsukura Y, Kamiie J, Terasaki T. Quantitative membrane protein expression at the blood-brain barrier of adult and younger cynomolgus monkeys. J Pharm Sci. 2011;100(9):3939-3950.

4. Hoshi Y, Uchida Y, Tachikawa M, Inoue T, Ohtsuki S, Terasaki T. Quantitative atlas of blood-brain barrier transporters, receptors, and tight junction proteins in rats and common marmoset. J Pharm Sci. 2013;102(9):3343-3355.

5. Hammarlund-Udenaes M, Friden M, Syvanen S, Gupta A. On the rate and extent of drug delivery to the brain. Pharm Res. 2008;25(8):1737-1750.

6. Knibbe CA, Zuideveld KP, Aarts LP, Kuks PF, Danhof M. Allometric relationships between the pharmacokinetics of propofol in rats, children and adults. Br J Clin Pharmacol. 2005;59(6):705-711.

7. Rais R, Thomas AG, Wozniak K, Wu Y, Jaaro-Peled H, Sawa A, Strick CA, Engle SJ, Brandon NJ, Rojas C, Slusher BS, Tsukamoto T. Pharmacokinetics of oral D-serine in D-amino acid oxidase knockout mice. Drug Metab Dispos. 2012;40(11):2067-2073.

8. Kantrowitz JT, Malhotra AK, Cornblatt B, Silipo G, Balla A, Suckow RF, D'Souza C, Saksa J, Woods SW, Javitt DC. High dose D-serine in the treatment of schizophrenia. Schizophr Res. 2010;121(1-3):125-130.
