# Supplementary material for: Mechanistic Multilayer Quantitative Model for Nonlinear Pharmacokinetics, Target Occupancy and Pharmacodynamics (PK/TO/PD) Relationship of D-Amino Acid Oxidase Inhibitor, TAK-831 in Mice
Source: Pharm Res. 2020 Aug 5;37(8):164. doi: 10.1007/s11095-020-02893-x (PMC7478952; doi:10.1007/s11095-020-02893-x)
Supplement: Supplementary file 2 — (DOCX 77 kb) [file 11095_2020_2893_MOESM2_ESM.docx]

**Supplementary table 1** Summary of group allocation of C57BL/6J mice in preclinical studies of TAK-831

| Study type | Dose (mg/kg) | Time (h) | | | | | |
| --- | --- | --- | --- | --- | --- | --- | --- |
|  |  | 2 | 4 | 6 | 8 | 10 | 24 |
| PK/TO | 0 | n=4 | n=4 | n=4 | n=4 | n=4 | n=4 |
|  | 0.3 | n=4 | n=4 | n=4 | n=4 | n=4 | n=4 |
|  | 1 | n=4 | n=4 | n=4 | n=4 | n=4 | n=4 |
|  | 3 | n=4 | n=4 | n=4 | n=4 | n=4 | n=4 |
|  | 10 | n=4 | n=4 | n=4 | n=4 | n=4 | n=4 |
|  | Total n=120 | | | | | | |

| Study type | Dose (mg/kg) | Time (h) | | | |
| --- | --- | --- | --- | --- | --- |
|  |  | 2 | 6 | 10 | 24 |
| PD | 0 | n=7 | n=7 | n=7 | n=7 |
|  | 0.3 | n=7 | n=7 | n=7 | n=7 |
|  | 1 | n=7 | n=7 | n=7 | n=7 |
|  | 3 | n=7 | n=7 | n=7 | n=7 |
|  | 10 | n=7 | n=7 | n=7 | n=7 |
|  | Total n=140 | | | | |

| Study type | Dose (mg/kg) | Time (h) | | | | | | |
| --- | --- | --- | --- | --- | --- | --- | --- | --- |
|  |  | 0.25 | 0.5 | 1 | 2 | 4 | 8 | 24 |
| Brain distribution | 0.3 | n=2 | n=2 | n=2 | n=2 | n=2 | n=2 | n=2 |
|  | 3 | n=2 | n=2 | n=2 | n=2 | n=2 | n=2 | n=2 |
|  | Total n=28 | | | | | | | |

| Study type | Dose (mg/kg) | Time (h) | | |
| --- | --- | --- | --- | --- |
|  |  | 0.25 | 1 | 6 |
| Metabolite profiling | 3 | n=2 | n=2 | n=2 |
|  | Total n=6 | | | |

PK, pharmacokinetics; TO, target occupancy; PD, pharmacodynamic
