# Supplementary material for: Mechanistic Multilayer Quantitative Model for Nonlinear Pharmacokinetics, Target Occupancy and Pharmacodynamics (PK/TO/PD) Relationship of D-Amino Acid Oxidase Inhibitor, TAK-831 in Mice
Source: Pharm Res. 2020 Aug 5;37(8):164. doi: 10.1007/s11095-020-02893-x (PMC7478952; doi:10.1007/s11095-020-02893-x)
Supplement: Supplementary file 3 — (DOCX 76 kb) [file 11095_2020_2893_MOESM3_ESM.docx]

Supplementary Table S2 The tabulated model parameters for translated PK/TO/PD of TAK-831 in humans

| Category | Parameter | Unit | Definition | Value |
| --- | --- | --- | --- | --- |
| PK | CL | L/h/kg | plasma clearance | 0.101 |
|  | V1 | L/kg | central volume of distribution | 0.0846 |
|  | Q | L/h/kg | inter-compartmental clearance | 0.0613 |
|  | V2 | L/kg | peripheral volume of distribution | 1.34 |
|  | Ka | 1/h | absorption rate constant | 0.218 |
|  | F | - | bioavailability | 0.334 |
| TO | Kon | mL/ng/h | association rate constant | 0.00324 |
|  | Koff | 1/h | dissociation rate constant | 0.113 |
|  | BRmax | - | maximum binding ratio | 0.987 |
| PD | BL | - | baseline of D-serine | 1 |
|  | Kout | 1/h | elimination rate constant | 0.480 |
|  | Imax | - | maximum inhibitory effect | 0.877 |

-: not applicable; PK, pharmacokinetics; TO, target occupancy; PD, pharmacodynamics
