# Supplementary figures and images for: Mechanistic Multilayer Quantitative Model for Nonlinear Pharmacokinetics, Target Occupancy and Pharmacodynamics (PK/TO/PD) Relationship of D-Amino Acid Oxidase Inhibitor, TAK-831 in Mice
Source: Pharm Res. 2020 Aug 5;37(8):164. doi: 10.1007/s11095-020-02893-x (PMC7478952; doi:10.1007/s11095-020-02893-x)

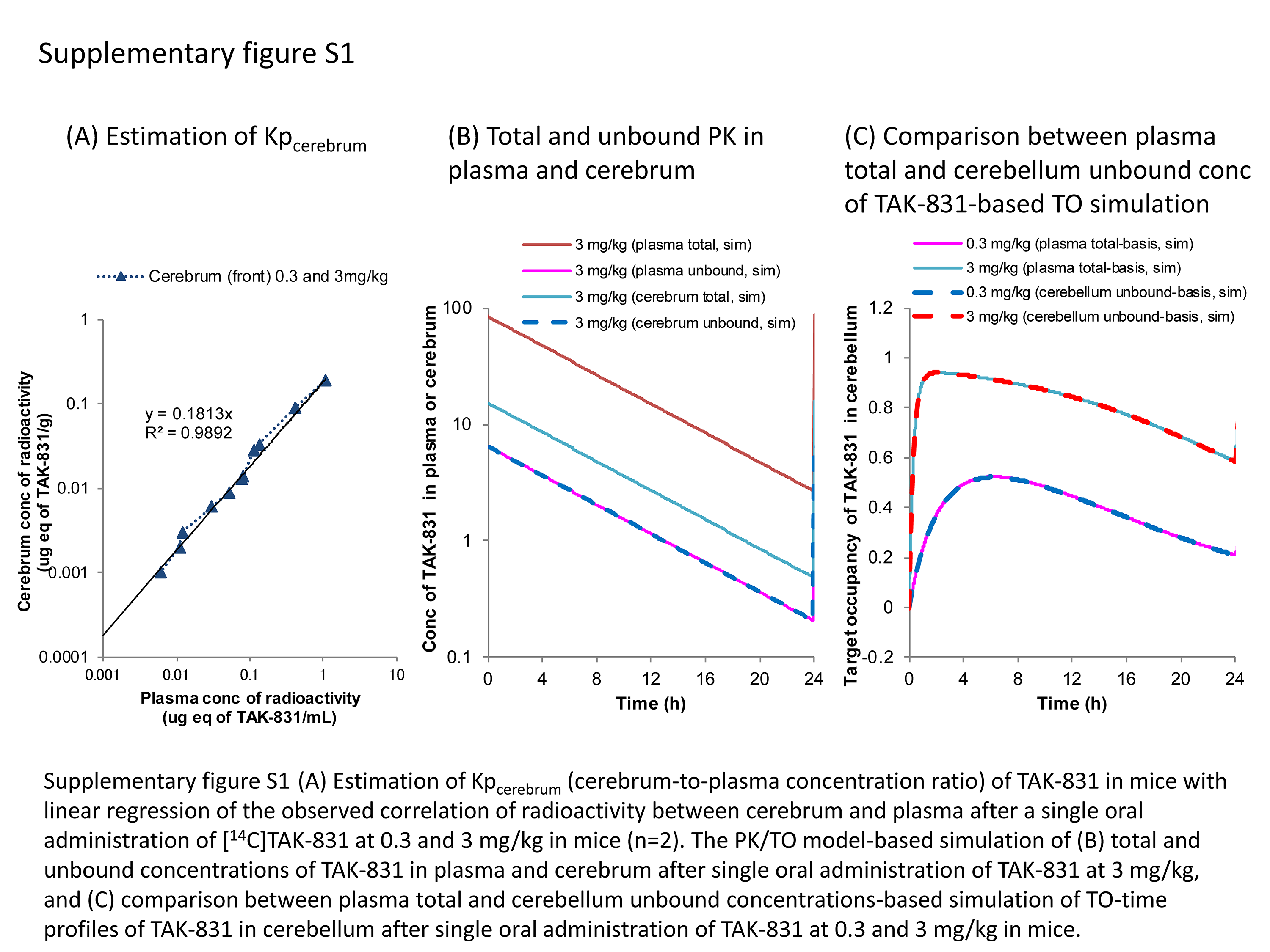

Supplement: Supplementary file 4 — (PNG 580 kb) [file 11095_2020_2893_Fig6_ESM.png]

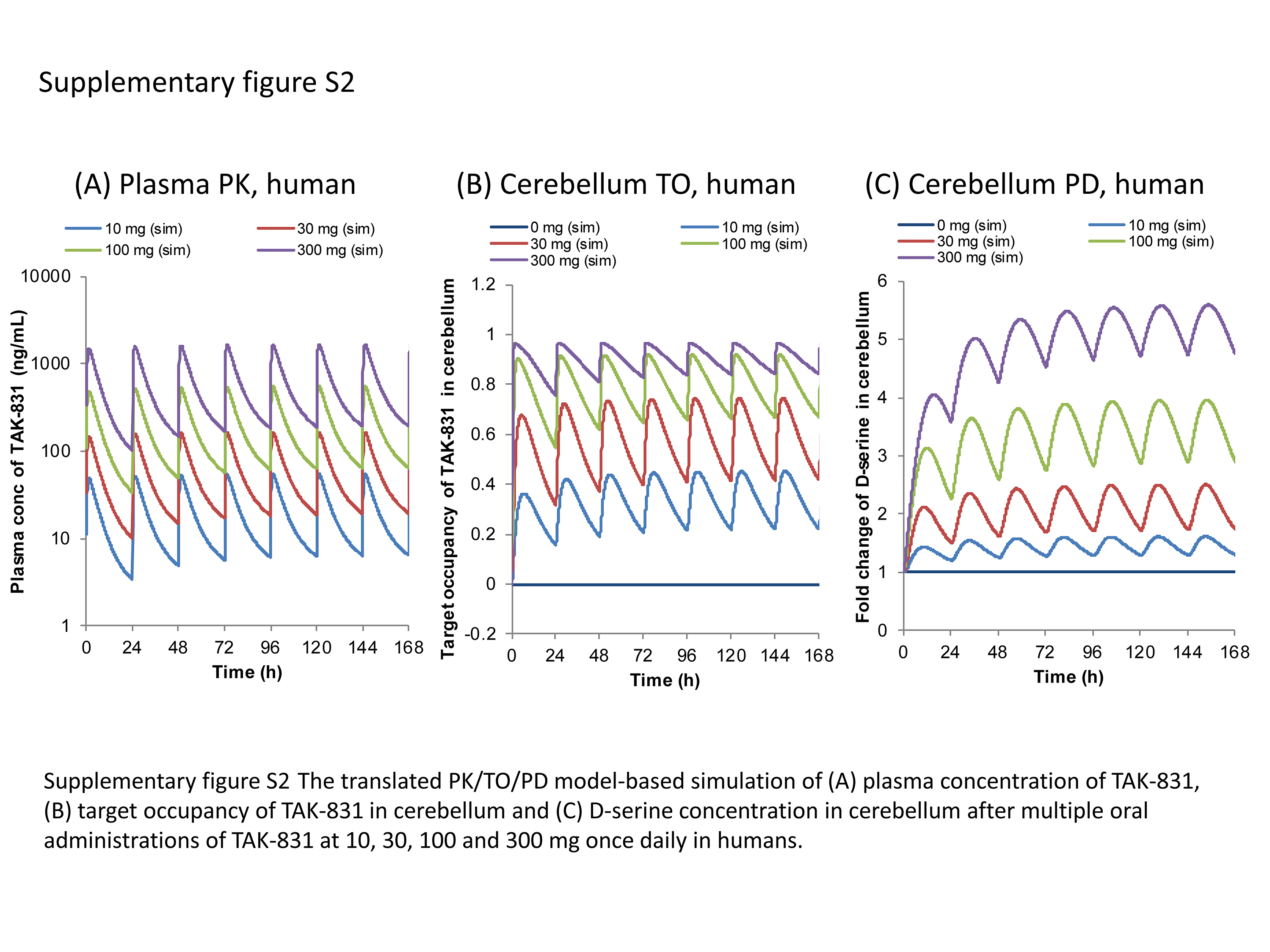

Supplement: Supplementary file 6 — (PNG 658 kb) [file 11095_2020_2893_Fig7_ESM.png]
